# Hospitalization for Chagas Heart Disease in the United States From 2002 to 2017

**DOI:** 10.1001/jamanetworkopen.2021.29959

**Published:** 2021-10-19

**Authors:** Neiberg de Alcantara Lima, David T. Martin, Ricardo Lessa de Castro, Adam Ladzinski, Anya Ring, Duncan Vos, Thomas A. Melgar

**Affiliations:** 1 Department of Internal Medicine, Western Michigan University Homer Stryker M.D. School of Medicine, Kalamazoo; 2 Department of Internal Medicine, Division of Cardiology, Wayne State University, Detroit, Michigan; 3 Department of Medicine, Brigham and Women’s Hospital, Harvard Medical School, Boston, Massachusetts; 4 Division of Cardiology, John H. Stroger, Jr. Hospital of Cook County, Chicago, Illinois; 5 Department of Pediatrics, Adolescent and Internal Medicine, Western Michigan University Homer Stryker M.D. School of Medicine, Kalamazoo.; 6 Department of Statistics, Western Michigan University Homer Stryker M.D. School of Medicine, Kalamazoo

## Abstract

This cross-sectional study examines characteristics and outcomes of estimated discharges for Chagas heart disease in the United States from 2002 to 2017.

## Introduction

Chagas disease is caused by the parasitic flagellate protozoa *Trypanosoma cruzi* and can affect multiple organs, including the heart.^1^ In the United States, most patients with Chagas cardiomyopathy are immigrants from regions in Central and South America where the disease is endemic.^2^ In this cross-sectional study, we sought to describe patient characteristics and outcomes of patients hospitalized for Chagas cardiomyopathy in the United States and trends in the percentage of hospital discharges for Chagas cardiomyopathy.

## Methods

This cross-sectional study was deemed exempt from protocol review and informed consent by our institutional review board owing to the deidentified nature of the data set. This study is reported following the Strengthening the Reporting of Observational Studies in Epidemiology (STROBE) reporting guideline.

Our study population included patients discharged with the diagnosis of Chagas heart disease in the United States from 2002 through 2017 (except 2015) using data from the Healthcare Cost and Utilization Project National Inpatient Sample (HCUP-NIS). Survey procedures using discharge weights provided with the HCUP-NIS database were used to generate national estimates. The HCUP-NIS database contains data on approximately 482 million inpatient discharges for the study period. Ethnicity was self-reported or determined by clerk at admission. As Chagas heart disease is endemic in South and Central America, previous literature has shown racial and ethnic disparities in the incidence in the US.

Diagnoses were determined using *International Classification of Diseases, Ninth Revision* and *International Statistical Classification of Diseases and Related Health Problems, Tenth Revision *codes (eAppendix in the Supplement)*.* Coded commonly related concurrent conditions in patients with Chagas heart disease, including node dysfunction, and surgical procedures, such as pacemaker placement, were evaluated.

SAS Studio (SAS Institute) was used for all analyses, and all tests were 2-sided. The prevalence of CHD was assessed by calculating a 95% CI of the observed proportion. To assess the trend of the frequency of Chagas heart disease over time, Cochran-Armitage trend tests were performed. Statistical significance was set a α = .05 to protect against the increased probability of type I errors that result from multiple testing. Data were analyzed in January 2020.

## Results

Chagas heart disease was coded as a primary or a coexisting diagnosis in a total of 2037 estimated discharges (mean age, 51.84 [26.63-71.22] years; 1028 [50.5%] men and 1009 [49.5%] women). Of 1744 estimated Chagas heart disease discharges in which a hospital region was indicated, 625 (35.9%) were diagnosed in the West region, and 564 (32.3%) were from the South region. Hispanic patients represent 12.8% of overall hospital discharges in the US, but 1433 estimated discharges (74.2%) for Chagas heart disease were patients of Hispanic ethnicity.

Sinoatrial node dysfunction was coded in 97 discharges (4.8%), atrial tachyarrhythmias were coded in 553 discharges (27.1%), and ventricular arrhythmias were coded in 509 discharges (25.0%). Atrioventricular and intraventricular conduction anomalies were common in patients with Chagas heart disease (553 discharges [14.5%]). There were 1316 estimated discharges (64.6%) that also had a diagnostic code for heart failure. Pacemaker implant procedure codes were reported in 51 discharges (2.5%), implantable defibrillator procedure codes in 219 discharges (10.7%), and cardiac resynchronization procedure codes in 59 discharges (2.9%) (Table). The proportion of hospital discharges with Chagas heart disease increased from 2002 to 2017 (Figure).

**Table.** General Characteristics, Concurrent Conditions, and Procedures Performed in Estimated Discharges for Chagas Heart Disease

Characteristic	Estimated discharges, No. (%) (N = 2037)
Age, mean (95% CI), y	51.84 (26.63-71.22)
Sex	
Men	1028 (50.5)
Women	1009 (49.5)
In-hospital mortality, No. (%) [95% CI]^a^	54 (2.7) [1.1-4.2]
Race and ethnicity	
African American, Asian, Pacific Islander, Native American, or other^b^	240 (12.4)
Hispanic	1433 (74.2)
White	259 (13.4)
Payer	
Medicare	561 (27.6)
Medicaid	676 (33.3)
Private insurance	359 (17.7)
No charge	90 (4.4)
Self pay or other	345 (17.0)
Region	
Northeast	436 (25.0)
Midwest	119 (6.8)
South	564 (32.3)
West	625 (35.8)
Concurrent conditions, No. (%) [95% CI]^a^	
Pulmonary hypertension	211 (10.3) [7.4-13.3]
Sinoatrial dysfunction	97 (4.8) [2.7-6.8]
Atrioventricular Node block intraventricular conduction abnormalities	95 (14.5) [8.8-20.2]
Atrial tachyarrhythmias	553 (27.1) [21.8-32.5]
Ventricular arrhythmias	509 (25.0) [20.6-29.4]
Heart failure	1316 (64.6) [59.8-69.5]
Stroke	100 (4.9) [2.5-7.2]
Procedure performed	
Pacemaker placement	51 (2.5)
EP study or ablation	73 (3.6)
Cardiac resynchronization therapy	59 (2.9)
Implantable cardioverter defibrillator	219 (10.7)

Abbreviations: EP, electrophysiology.

^a^ We include 95% CIs for proportions of interest, which includes all proportions for co-occurring comorbidities.

^b^ Other race and ethnicity includes individuals who self-reported as other race or ethnicity (ie, not African American, Asian, Hispanic, Native American, Pacific Islander, or White).

**Figure.**
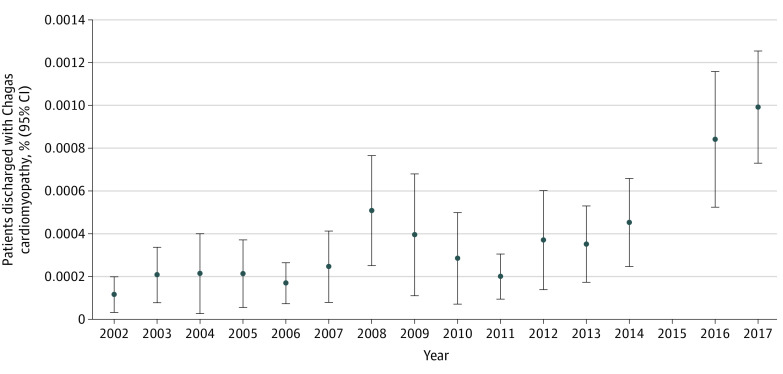
Trend of Chagas Heart Disease Hospitalizations From 2002-2017 The Cochran-Armitage trend test indicated there was a significant uptrend (*P* < .001). Since 2015 was the year of transition between *International Classification of Diseases, Ninth Revision,* and *International Statistical Classification of Diseases and Related Health Problems, Tenth Revision*, which could cause inconsistencies in reporting, this year was not included in analysis.

## Discussion

This cross-sectional study found that among the 482 million estimated discharges over 12 years in the HCUP-NIS data set, only 2037 estimated discharges were coded for Chagas heart disease. Previous reports based on patterns of emigration from South America have estimated a prevalence of Chagas as 300 000 cases in the US, 3000 cases in Canada, and 123 000 cases in Europe.^3,4,5^ Our findings suggest that hospitalizations for Chagas heart disease have increased every year of our study and that Chagas heart disease is more prevalent in the West and South regions of the US, where migration from South American is higher.

Arrhythmias are common in Chagas heart disease.^6^ This was evident in our study, as 27.1% of discharges had atrial tachyarrhythmias and 25.0% of discharges had ventricular tachyarrhythmias. Device management was common, as 2.5% of discharges had pacemaker placement and nearly 11% of discharges had an implantable cardiac defibrillator placed.

This study has some limitations. The NIS-HCUP database does not provide individual patient-level information, and only diagnostic codes used during the hospitalization are included; this may result in underrepresentation of Chagas disease. Multiple hospitalizations of a single patient could have been counted. Since 2015 was the year of transition between *ICD-9* and *ICD-10,* which could cause inconsistencies in reporting, this year was not included in our study.

Using data from the HCUP-NIS, this cross-sectional study described patient characteristics and outcomes of patients discharged with a diagnosis of Chagas heart disease and found a temporal increase in the proportion of hospital discharges for Chagas heart disease in the US. Recognition of this condition, particularly in individuals who emigrated from areas with endemic Chagas, may assist and early diagnosis and avoidance of long-term complications from Chagas heart disease.

## Notes

Supplement. **eAppendix.** *ICD-9* and *ICD-10* Codes
