# Supplementary material for: Hospitalization for Chagas Heart Disease in the United States From 2002 to 2017
Source: JAMA Netw Open. 2021 Oct 19;4(10):e2129959. doi: 10.1001/jamanetworkopen.2021.29959 (PMC8527354; doi:10.1001/jamanetworkopen.2021.29959)
Supplement: Supplement. — eAppendix. ICD-9 and ICD-10 Codes [file jamanetwopen-e2129959-s001.pdf]

Supplemental Online Content

Lima NDA, Martin DT, de Castro RL Jr, et al. Hospitalization for Chagas heart disease in the United States from 2002 to 2017. *JAMA Netw Open*. 2021;4(10):e2129959. doi:10.1001/jamanetworkopen.2021.29959

eAppendix. *ICD-9* and *ICD-10* Codes

This supplemental material has been provided by the authors to give readers additional information about their work.

© 2021 de Alcantara Lima N et al. *JAMA Network Open.*

eAppendix. *ICD-9* and *ICD-10* Codes

ICD 9 CODES USED

Chagas Cardiomyopathy
086.0 Chagas disease with heart involvement

Heart Failure Codes
428.0Congestive heart failure, unspecified
428.1Left heart failure
428.2 Systolic heart failure
428.20Systolic heart failure, unspecified
428.21Acute systolic heart failure
428.22Chronic systolic heart failure
428.23Acute on chronic systolic heart failure
428.4 Combined systolic and diastolic heart failure
428.40Combined systolic and diastolic heart failure, unspecified
428.41Acute combined systolic and diastolic heart failure
428.42Chronic combined systolic and diastolic heart failure
428.43Acute on chronic combined systolic and diastolic heart failure

514 **Pulmonary** congestion and hypostasis

518.4 - Acute edema of lung, unspecified

Pulmonary Hypertension codes
416.8Other chronic pulmonary heart diseases
416.9Chronic pulmonary heart disease, unspecified

Stroke
438 Late effects of cerebrovascular disease
436 - Cva
V12.54 - Hx TIA/stroke w/o resid
434 Occlusion of cerebral arteries
434.9 Cerebral artery occlusion, unspecified
434.90Cerebral artery occlusion, unspecified without mention of cerebral infarction
434.91Cerebral artery occlusion, unspecified with cerebral infarction
435.9 - Trans cereb ischemia NOS

Atrial Tachyarrhythmias
427.31 - Atrial fibrillation
427.3 Atrial fibrillation and flutter
427.32 - Atrial flutter

© 2021 de Alcantara Lima N et al. *JAMA Network Open.*

427.0 - Parox atrial tachycardia
427.61 Supraventricular premature beats

Sinoatrial Node Dysfunction
427.81 Sinoatrial node dysfunction

Severe AV Node Block
426.0 Atrioventricular block, complete
426.12 Mobitz (type) II atrioventricular block
426.13 Other second degree atrioventricular block

Other EKG abnormalities

426.10 Atrioventricular block, unspecified
426.11 First degree atrioventricular block
426.2 Left bundle branch hemiblock
426.3 Other left bundle branch block
426.4 Right bundle branch block
426.5 Bundle branch block other and unspecified
426.50 Bundle branch block, unspecified
426.51 Right bundle branch block and left posterior fascicular block
426.52 Right bundle branch block and left anterior fascicular block
426.53 Other bilateral bundle branch block
426.54 Trifascicular block

Syncope
780.2 - Syncope and collapse

Ventricular Arrhythmias
427.4 Ventricular fibrillation and flutter
427.41Ventricular fibrillation
427.42Ventricular flutter
427.60 Premature beats, unspecified
427.1 Paroxysmal ventricular tachycardia

Cardiac Arrest
427.5 Cardiac arrest

Arterial Embolization

444.0 Embolism and thrombosis of abdominal aorta
444.01 Saddle embolus of abdominal aorta
444.09 Other arterial embolism and thrombosis of abdominal aorta
444.1 Embolism and thrombosis of thoracic aorta
444.2 Embolism and thrombosis of arteries of the extremities

© 2021 de Alcantara Lima N et al. *JAMA Network Open.*

444.21 Arterial embolism and thrombosis of upper extremity
444.22 Arterial embolism and thrombosis of lower extremity
444.8 Embolism and thrombosis of other specified artery
444.81 Embolism and thrombosis of iliac artery
444.89 Embolism and thrombosis of other specified
444.9 Embolism and thrombosis of unspecified artery

Electric Cardioversion
99.61Atrial **cardioversion**
99.62Other electric countershock of heart
99.69Other conversion of cardiac rhythm

Ablation / EP Study (Codes Still Pending???????)
37.26Catheter based invasive electrophysiologic testing
37.27Cardiac mapping
C1730 Catheter, electrophysiology, diagnostic, other than 3d mapping (19 or fewer...
C1731 Catheter, electrophysiology, diagnostic, other than 3d mapping (20 or more
C1732 Catheter, electrophysiology, diagnostic/ablation, 3d or vector mappingC1733 Catheter, electrophysiology, diagnostic/ablation, other than 3d or vector m…

Pacemaker placement
37.80Insertion of permanent pacemaker, initial or replacement, type of device not specified
37.81Initial insertion of single-chamber device, not specified as rate responsive
37.82Initial insertion of single-chamber device, rate responsive
37.83Initial insertion of dual-chamber device
37.85Replacement of any type pacemaker device with single-chamber device, not specified as rate responsive
37.86Replacement of any type of pacemaker device with single-chamber device, rate responsive
37.87Replacement of any type pacemaker device with dual-chamber device

CRT placement
00.50Implantation of cardiac resynchronization pacemaker without mention of defibrillation, total system [**CRT**-P]
00.51Implantation of cardiac resynchronization defibrillator, total system [**CRT**-D]
00.52Implantation or replacement of transvenous lead [electrode] into left ventricular coronary venous system
00.53Implantation or replacement of cardiac resynchronization pacemaker pulse generator only [**CRT**-P]
00.54Implantation or replacement of cardiac resynchronization defibrillator pulse generator only [**CRT**-D]

ICD placement

© 2021 de Alcantara Lima N et al. *JAMA Network Open.*

37.94Implantation or replacement of automatic cardioverter/**defibrillator**, total system [AICD]
37.95Implantation of automatic cardioverter/**defibrillator**lead(s) only
37.96Implantation of automatic cardioverter/**defibrillator**pulse generator only
37.97Replacement of automatic cardioverter/**defibrillator**lead(s) only
37.98Replacement of automatic cardioverter/**defibrillator**pulse generator only
00.51Implantation of cardiac resynchronization defibrillator, total system [**CRT**-D]
00.54Implantation or replacement of cardiac resynchronization defibrillator pulse generator only [**CRT**-D]

Heart Transplant
37.51Heart transplantation

Circulatory support devices Placement

37.52Implantation of total internal biventricular **heart**replacement system
37.53Replacement or repair of thoracic unit of (total) replacement **heart** system
37.54Replacement or repair of other implantable component of (total) replacement **heart** system
37.60Implantation or insertion of biventricular external heart assist system
37.61Implant of pulsation **balloon**
37.62Insertion of temporary non-implantable extracorporeal circulatory assist device
37.63Repair of heart assist system
37.65Implant of single ventricular (extracorporeal) external heart assist system
37.66Insertion of implantable heart assist system
37.68Insertion of percutaneous external heart assist device
39.65Extracorporeal membrane oxygenation [ECMO]

SUPPLEMENT
ICD 10 CODES USED

Chagas Cardiomyopathy

B570 ACUTE CHAGAS' DISEASE WITH HEART INVOLVEMENT
B572 CHAGAS' DISEASE (CHRONIC) WITH HEART INVOLVEMENT

Heart Failure Codes

I509 - HEART FAILURE, UNSPECIFIED
I501 - LEFT VENTRICULAR FAILURE
I5020 - UNSPECIFIED SYSTOLIC (CONGESTIVE) HEART FAILURE
I5021 ACUTE SYSTOLIC (CONGESTIVE) HEART FAILURE
I5022 - CHRONIC SYSTOLIC (CONGESTIVE) HEART FAILURE
I5023 - ACUTE ON CHRONIC SYSTOLIC (CONGESTIVE) HEART FAILURE
I5040 - UNSP COMBINED SYSTOLIC AND DIASTOLIC (CONGESTIVE) HRT FAIL
I5041 - ACUTE COMBINED SYSTOLIC AND DIASTOLIC (CONGESTIVE) HRT FAIL

© 2021 de Alcantara Lima N et al. *JAMA Network Open.*

I5042 - CHRONIC COMBINED SYSTOLIC AND DIASTOLIC HRT FAIL
I5043 - ACUTE ON CHRONIC COMBINED SYSTOLIC AND DIASTOLIC HRT FAIL
J810 - ACUTE PULMONARY EDEMA
J811 - CHRONIC PULMONARY EDEMA
I50.3 Diastolic (congestive) heart failure
I50.30 Unspecified diastolic (congestive) heart failure
I50.31 Acute diastolic (congestive) heart failure
I50.32 Chronic diastolic (congestive) heart failure
I50.33 Acute on chronic diastolic (congestive) heart failure
I50.8 Other heart failure
I50.81 Right heart failure
I50.810 …… unspecified
I50.811 Acute right heart failure
I50.812 Chronic right heart failure
I50.813 Acute on chronic right heart failure
I50.814 …… dueo left heart failure
I50.82 Biventricular heart failure
I50.83 High output heart failure
I50.84 End stage heart failure
I50.89 Other heart failure
I50.9 Heart failure, unspecified

Pulmonary Hypertension codes

I272 OTHER SECONDARY PULMONARY HYPERTENSION
I2789 OTHER SPECIFIED PULMONARY HEART DISEASES
I2781 COR PULMONALE (CHRONIC)
I279 PULMONARY HEART DISEASE, UNSPECIFIED
I27.0 Primary pulmonary hypertension
I27.20 Pulmonary hypertension, unspecified
I27.21 Secondary pulmonary arterial hypertension
I27.22 Pulmonary hypertension due to left heart disease
I27.8 Other specified pulmonary heart diseases

Stroke

I63.0 Cerebral infarction due to thrombosis of precerebral arteries
I63.00 Cerebral infarction due to thrombosis of unspecified precerebral artery
I63.01 Cerebral infarction due to thrombosis of vertebral artery
I63.011 Cerebral infarction due to thrombosis of right vertebral artery
I63.012 Cerebral infarction due to thrombosis of left vertebral artery
I63.013 Cerebral infarction due to thrombosis of bilateral vertebral arteries
I63.019 Cerebral infarction due to thrombosis of unspecified vertebral artery
I63.02 Cerebral infarction due to thrombosis of basilar artery
I63.03 Cerebral infarction due to thrombosis of carotid artery
I63.031 Cerebral infarction due to thrombosis of right carotid artery
I63.032 Cerebral infarction due to thrombosis of left carotid artery
I63.033 Cerebral infarction due to thrombosis of bilateral carotid arteries
I63.039 Cerebral infarction due to thrombosis of unspecified carotid artery
I63.09 Cerebral infarction due to thrombosis of other precerebral artery

© 2021 de Alcantara Lima N et al. *JAMA Network Open.*

I63.1 Cerebral infarction due to embolism of precerebral arteries
I63.10 Cerebral infarction due to embolism of unspecified precerebral artery
I63.11 Cerebral infarction due to embolism of vertebral artery
I63.111 Cerebral infarction due to embolism of right vertebral artery
I63.112 Cerebral infarction due to embolism of left vertebral artery
I63.113 Cerebral infarction due to embolism of bilateral vertebral arteries
I63.119 Cerebral infarction due to embolism of unspecified vertebral artery
I63.12 Cerebral infarction due to embolism of basilar artery
I63.13 Cerebral infarction due to embolism of carotid artery
I63.131 Cerebral infarction due to embolism of right carotid artery
I63.132 Cerebral infarction due to embolism of left carotid artery
I63.133 Cerebral infarction due to embolism of bilateral carotid arteries
I63.139 Cerebral infarction due to embolism of unspecified carotid artery
I63.19 Cerebral infarction due to embolism of other precerebral artery
I63.2 Cerebral infarction due to unspecified occlusion or stenosis of precerebral arteries
I63.20 Cerebral infarction due to unspecified occlusion or stenosis of unspecified precerebral arteries
I63.21 Cerebral infarction due to unspecified occlusion or stenosis of vertebral arteries
I63.211 Cerebral infarction due to unspecified occlusion or stenosis of right vertebral artery
I63.212 Cerebral infarction due to unspecified occlusion or stenosis of left vertebral artery
I63.213 Cerebral infarction due to unspecified occlusion or stenosis of bilateral vertebral arteries
I63.219 Cerebral infarction due to unspecified occlusion or stenosis of unspecified vertebral artery
I63.22 Cerebral infarction due to unspecified occlusion or stenosis of basilar artery
I63.23 Cerebral infarction due to unspecified occlusion or stenosis of carotid arteries
I63.231 Cerebral infarction due to unspecified occlusion or stenosis of right carotid arteries
I63.232 Cerebral infarction due to unspecified occlusion or stenosis of left carotid arteries
I63.233 Cerebral infarction due to unspecified occlusion or stenosis of bilateral carotid arteries
I63.239 Cerebral infarction due to unspecified occlusion or stenosis of unspecified carotid artery
I63.29 Cerebral infarction due to unspecified occlusion or stenosis of other precerebral arteries
I63.3 Cerebral infarction due to thrombosis of cerebral arteries
I63.30 Cerebral infarction due to thrombosis of unspecified cerebral artery
I63.31 Cerebral infarction due to thrombosis of middle cerebral artery
I63.311 Cerebral infarction due to thrombosis of right middle cerebral artery
I63.312 Cerebral infarction due to thrombosis of left middle cerebral artery
I63.313 Cerebral infarction due to thrombosis of bilateral middle cerebral arteries
I63.319 Cerebral infarction due to thrombosis of unspecified middle cerebral artery
I63.32 Cerebral infarction due to thrombosis of anterior cerebral artery
I63.321 Cerebral infarction due to thrombosis of right anterior cerebral artery
I63.322 Cerebral infarction due to thrombosis of left anterior cerebral artery
I63.323 Cerebral infarction due to thrombosis of bilateral anterior cerebral arteries
I63.329 Cerebral infarction due to thrombosis of unspecified anterior cerebral artery
I63.33 Cerebral infarction due to thrombosis of posterior cerebral artery
I63.331 Cerebral infarction due to thrombosis of right posterior cerebral artery
I63.332 Cerebral infarction due to thrombosis of left posterior cerebral artery
I63.333 Cerebral infarction due to thrombosis of bilateral posterior cerebral arteries
I63.339 Cerebral infarction due to thrombosis of unspecified posterior cerebral artery

© 2021 de Alcantara Lima N et al. *JAMA Network Open.*

I63.34 Cerebral infarction due to thrombosis of cerebellar artery
I63.341 Cerebral infarction due to thrombosis of right cerebellar artery
I63.342 Cerebral infarction due to thrombosis of left cerebellar artery
I63.343 Cerebral infarction due to thrombosis of bilateral cerebellar arteries
I63.349 Cerebral infarction due to thrombosis of unspecified cerebellar artery
I63.39 Cerebral infarction due to thrombosis of other cerebral artery
I63.4 Cerebral infarction due to embolism of cerebral arteries
I63.40 Cerebral infarction due to embolism of unspecified cerebral artery
I63.41 Cerebral infarction due to embolism of middle cerebral artery
I63.411 Cerebral infarction due to embolism of right middle cerebral artery
I63.412 Cerebral infarction due to embolism of left middle cerebral artery
I63.413 Cerebral infarction due to embolism of bilateral middle cerebral arteries
I63.419 Cerebral infarction due to embolism of unspecified middle cerebral artery
I63.42 Cerebral infarction due to embolism of anterior cerebral artery
I63.421 Cerebral infarction due to embolism of right anterior cerebral artery
I63.422 Cerebral infarction due to embolism of left anterior cerebral artery
I63.423 Cerebral infarction due to embolism of bilateral anterior cerebral arteries
I63.429 Cerebral infarction due to embolism of unspecified anterior cerebral artery
I63.43 Cerebral infarction due to embolism of posterior cerebral artery
I63.431 Cerebral infarction due to embolism of right posterior cerebral artery
I63.432 Cerebral infarction due to embolism of left posterior cerebral artery
I63.433 Cerebral infarction due to embolism of bilateral posterior cerebral arteries
I63.439 Cerebral infarction due to embolism of unspecified posterior cerebral artery
I63.44 Cerebral infarction due to embolism of cerebellar artery
I63.441 Cerebral infarction due to embolism of right cerebellar artery
I63.442 Cerebral infarction due to embolism of left cerebellar artery
I63.443 Cerebral infarction due to embolism of bilateral cerebellar arteries
I63.449 Cerebral infarction due to embolism of unspecified cerebellar artery
I63.49 Cerebral infarction due to embolism of other cerebral artery
I63.5 Cerebral infarction due to unspecified occlusion or stenosis of cerebral arteries
I63.50 Cerebral infarction due to unspecified occlusion or stenosis of unspecified cerebral artery
I63.51 Cerebral infarction due to unspecified occlusion or stenosis of middle cerebral artery
I63.511 Cerebral infarction due to unspecified occlusion or stenosis of right middle cerebral artery
I63.512 Cerebral infarction due to unspecified occlusion or stenosis of left middle cerebral artery
I63.513 Cerebral infarction due to unspecified occlusion or stenosis of bilateral middle cerebral arteries
I63.519 Cerebral infarction due to unspecified occlusion or stenosis of unspecified middle cerebral artery
I63.52 Cerebral infarction due to unspecified occlusion or stenosis of anterior cerebral artery
I63.521 Cerebral infarction due to unspecified occlusion or stenosis of right anterior cerebral artery
I63.522 Cerebral infarction due to unspecified occlusion or stenosis of left anterior cerebral artery
I63.523 Cerebral infarction due to unspecified occlusion or stenosis of bilateral anterior cerebral arteries
I63.529 Cerebral infarction due to unspecified occlusion or stenosis of unspecified anterior cerebral artery

© 2021 de Alcantara Lima N et al. *JAMA Network Open.*

I63.53 Cerebral infarction due to unspecified occlusion or stenosis of posterior cerebral artery
I63.531 Cerebral infarction due to unspecified occlusion or stenosis of right posterior cerebral artery
I63.532 Cerebral infarction due to unspecified occlusion or stenosis of left posterior cerebral artery
I63.533 Cerebral infarction due to unspecified occlusion or stenosis of bilateral posterior cerebral arteries
I63.539 Cerebral infarction due to unspecified occlusion or stenosis of unspecified posterior cerebral artery
I63.54 Cerebral infarction due to unspecified occlusion or stenosis of cerebellar artery
I63.541 Cerebral infarction due to unspecified occlusion or stenosis of right cerebellar artery
I63.542 Cerebral infarction due to unspecified occlusion or stenosis of left cerebellar artery
I63.543 Cerebral infarction due to unspecified occlusion or stenosis of bilateral cerebellar arteries
I63.549 Cerebral infarction due to unspecified occlusion or stenosis of unspecified cerebellar artery
I63.59 Cerebral infarction due to unspecified occlusion or stenosis of other cerebral artery
I63.8 Other cerebral infarction
I63.81 …… due to occlusion or stenosis of small artery
I63.89 Other cerebral infarction
I63.9 Cerebral infarction, unspecified

Atrial Tachyarrhythmias

I4891 - UNSPECIFIED ATRIAL FIBRILLATION
I4892 - UNSPECIFIED ATRIAL FLUTTER
I470 – Re-entry ventricular arrhythmia
I479 - Paroxysmal tachycardia, unspecified
I471 - SUPRAVENTRICULAR TACHYCARDI
I491 - ATRIAL PREMATURE DEPOLARIZATION

Sinoatrial Node Dysfunction

I495 - SICK SINUS SYNDROME
R00.1 – SINUS BRADYCARDIA SINOATRIAL BRADYCARDIA

Severe AV Node Block + Other EKG abnormalities

I442 - ATRIOVENTRICULAR BLOCK, COMPLETE
I441 - ATRIOVENTRICULAR BLOCK, SECOND DEGREE
I4430 - UNSPECIFIED ATRIOVENTRICULAR BLOCK
I440 - ATRIOVENTRICULAR BLOCK, FIRST DEGREE
I444 - LEFT ANTERIOR FASCICULAR BLOCK
I445 - LEFT ANTERIOR FASCICULAR BLOCK

© 2021 de Alcantara Lima N et al. *JAMA Network Open.*

I4460 - UNSPECIFIED FASCICULAR BLOCK
I4469 - OTHER FASCICULAR BLOCK
I447 - LEFT BUNDLE-BRANCH BLOCK, UNSPECIFIED
I4510 - UNSPECIFIED RIGHT BUNDLE-BRANCH BLOCK
I4519 - Other right bundle-branch block
I45.5 Other specified heart block
I 4439 - OTHER ATRIOVENTRICULAR BLOCK
I454 NONSPECIFIC INTRAVENTRICULAR BLOCK
I452 - BIFASCICULAR BLOCK
I453 - TRIFASCICULAR BLOCK

Syncope
R55 - SYNCOPE AND COLLAPSE

Ventricular Arrhythmias
I490 Ventricular Fibrillation and Flutter
I4901 - VENTRICULAR FIBRILLATION
I4902 - VENTRICULAR FLUTTER
I4940 - UNSPECIFIED PREMATURE DEPOLARIZATION
I493 - Ventricular premature depolarization
I494.4 - OTHER PREMATURE DEPOLARIZATION
I494.9 - OTHER PREMATURE DEPOLARIZATION
I472 – VENTRICULAR TACHYCARDIA
I4949 - VENTRICULAR PREMATURE DEPOLARIZATION

Cardiac Arrest
I469 - CARDIAC ARREST, CAUSE UNSPECIFIED
I490 Ventricular Fibrillation and Flutter

Arterial Embolization
I7401 SADDLE EMBOLUS OF ABDOMINAL AORTA
I7409 OTHER ARTERIAL EMBOLISM AND THROMBOSIS OF ABDOMINAL AORT
I7411 EMBOLISM AND THROMBOSIS OF THORACIC AORTA
I742 EMBOLISM AND THROMBOSIS OF ARTERIES OF THE UPPER EXTREMITIES
I743 EMBOLISM AND THROMBOSIS OF ARTERIES OF THE LOWER EXTREMITIES
I745 EMBOLISM AND THROMBOSIS OF ILIAC ARTERY
I748 EMBOLISM AND THROMBOSIS OF OTHER ARTERIES
I749 EMBOLISM AND THROMBOSIS OF UNSPECIFIED ARTERY

Electric Cardioversion
5A2204Z Restoration of Cardiac Rhythm, Single

Ablation / EP Study
4A023FZ Measurement of Cardiac Rhythm, Percutaneous Approach
02K80ZZ Map Conduction Mechanism, Open Approach
02K83ZZ Map Conduction Mechanism, Percutaneous Approach
02K84ZZ Map Conduction Mechanism, Percutaneous Endoscopic Approach

© 2021 de Alcantara Lima N et al. *JAMA Network Open.*

02K8 Conduction Mechanism
02K80 Open
02K80Z No Device
02K83 Percutaneous
02K83Z No Device
02K83ZZ Map Conduction Mechanism, Percutaneous Approach
02K84 Percutaneous Endoscopic
02K84Z No Device
02K84ZZ Map Conduction Mechanism, Percutaneous Endoscopic Approach
4A0204 Electrical Activity
4A0204Z Measurement of Cardiac Electrical Activity, Open Approach
4A020C Rate
4A020CZ Measurement of Cardiac Rate, Open Approach
4A020F Rhythm
4A020FZ Measurement of Cardiac Rhythm, Open Approach
4A020PZ Measurement of Cardiac Action Currents, Open Approach
4A023 Percutaneous
4A0234 Electrical Activity
4A0234Z Measurement of Cardiac Electrical Activity, Percutaneous Approach
4A0239 Output
4A0239Z Measurement of Cardiac Output, Percutaneous Approach
4A023C Rate
4A023CZ Measurement of Cardiac Rate, Percutaneous Approach
4A023F Rhythm
4A023FZ Measurement of Cardiac Rhythm, Percutaneous Approach
4A027 Via Natural or Artificial Opening
4A0274 Electrical Activity
4A0274Z Measurement of Cardiac Electrical Activity, Via Natural or Artificial Opening
4A027CZ Measurement of Cardiac Rate, Via Natural or Artificial Opening
4A027F Rhythm
4A027FZ Measurement of Cardiac Rhythm, Via Natural or Artificial Opening

Pacemaker placement

0JH60PZ Insertion of Cardiac Rhythm Related Device into Chest Subcutaneous Tissue and Fascia, Open Approach
0JH63PZ Insertion of Cardiac Rhythm Related Device into Chest Subcutaneous Tissue and Fascia, Percutaneous Approach
0JH604 Pacemaker, Single Chamber
0JH604Z Insertion of Pacemaker, Single Chamber into Chest Subcutaneous Tissue and Fascia, Open Approach
0JH605 Pacemaker, Single Chamber Rate Responsive
0JH605Z Insertion of Pacemaker, Single Chamber Rate Responsive into Chest Subcutaneous Tissue and Fascia, Open Approach
0JH606 Pacemaker, Dual Chamber
0JH606Z Insertion of Pacemaker, Dual Chamber into Chest Subcutaneous Tissue and Fascia, Open Approach

© 2021 de Alcantara Lima N et al. *JAMA Network Open.*

0JH634 Pacemaker, Single Chamber
0JH634Z Insertion of Pacemaker, Single Chamber into Chest Subcutaneous Tissue and Fascia, Percutaneous Approach
0JH635 Pacemaker, Single Chamber Rate Responsive
0JH635Z Insertion of Pacemaker, Single Chamber Rate Responsive into Chest Subcutaneous Tissue and Fascia, Percutaneous Approach
0JH636 Pacemaker, Dual Chamber
0JH636Z Insertion of Pacemaker, Dual Chamber into Chest Subcutaneous Tissue and Fascia, Percutaneous Approach
0JH804 Pacemaker, Single Chamber
0JH804Z Insertion of Pacemaker, Single Chamber into Abdomen Subcutaneous Tissue and Fascia, Open Approach
0JH805 Pacemaker, Single Chamber Rate Responsive
0JH805Z Insertion of Pacemaker, Single Chamber Rate Responsive into Abdomen Subcutaneous Tissue and Fascia, Open Approach
0JH806 Pacemaker, Dual Chamber
0JH806Z Insertion of Pacemaker, Dual Chamber into Abdomen Subcutaneous Tissue and Fascia, Open Approach
0JH807 Cardiac Resynchronization Pacemaker Pulse Generator
0JH807Z Insertion of Cardiac Resynchronization Pacemaker Pulse Generator into Abdomen Subcutaneous Tissue and Fascia, Open Approach
0JH834 Pacemaker, Single Chamber
0JH834Z Insertion of Pacemaker, Single Chamber into Abdomen Subcutaneous Tissue and Fascia, Percutaneous Approach
0JH835 Pacemaker, Single Chamber Rate Responsive
0JH835Z Insertion of Pacemaker, Single Chamber Rate Responsive into Abdomen Subcutaneous Tissue and Fascia, Percutaneous Approach
0JH836 Pacemaker, Dual Chamber
0JH836Z Insertion of Pacemaker, Dual Chamber into Abdomen Subcutaneous Tissue and Fascia, Percutaneous Approach
0JH837 Cardiac Resynchronization Pacemaker Pulse Generator
0JH837Z Insertion of Cardiac Resynchronization Pacemaker Pulse Generator into Abdomen Subcutaneous Tissue and Fascia, Percutaneous Approach
0JH838 Defibrillator Generator

0JH80PZ Insertion of Cardiac Rhythm Related Device into Abdomen Subcutaneous Tissue and Fascia, Open Approach
0JH83PZ Insertion of Cardiac Rhythm Related Device into Abdomen Subcutaneous Tissue and Fascia, Percutaneous Approach

CRT placement

02H40JZ Insertion of Pacemaker Lead into Coronary Vein, Open Approach
02H43JZ Insertion of Pacemaker Lead into Coronary Vein, Percutaneous Approach
02H44JZ Insertion of Pacemaker Lead into Coronary Vein, Percutaneous Endoscopic Approach
0JH607Z Insertion of Cardiac Resynchronization Pacemaker Pulse Generator into Chest Subcutaneous Tissue and Fascia, Open Approach
0JH637Z Insertion of Cardiac Resynchronization Pacemaker Pulse Generator into Chest Subcutaneous Tissue and Fascia, Percutaneous Approach
0JH807Z Insertion of Cardiac Resynchronization Pacemaker Pulse Generator into Abdomen Subcutaneous Tissue and Fascia, Open Approach

© 2021 de Alcantara Lima N et al. *JAMA Network Open.*

0JH837Z Insertion of Cardiac Resynchronization Pacemaker Pulse Generator into Abdomen Subcutaneous Tissue and Fascia, Percutaneous Approach
0JH609Z Insertion of Cardiac Resynchronization Defibrillator Pulse Generator into Chest Subcutaneous Tissue and Fascia, Open Approach
0JH609 Cardiac Resynchronization Defibrillator Pulse Generator
0JH637 Cardiac Resynchronization Pacemaker Pulse Generator
0JH639 Cardiac Resynchronization Defibrillator Pulse Generator
0JH809 Cardiac Resynchronization Defibrillator Pulse Generator
0JH839 Cardiac Resynchronization Defibrillator Pulse Generator
0JH639Z Insertion of Cardiac Resynchronization Defibrillator Pulse Generator into Chest Subcutaneous Tissue and Fascia, Percutaneous Approach
0JH809Z Insertion of Cardiac Resynchronization Defibrillator Pulse Generator into Abdomen Subcutaneous Tissue and Fascia, Open Approach
0JH839Z Insertion of Cardiac Resynchronization Defibrillator Pulse Generator into Abdomen Subcutaneous Tissue and Fascia, Percutaneous Approach

ICD placement

PCS 0JH608Z Insertion of Defibrillator Generator into Chest Subcutaneous Tissue and Fascia, Open Approach
0JH638Z Insertion of Defibrillator Generator into Chest Subcutaneous Tissue and Fascia, Percutaneous Approach
0JH608 Defibrillator Generator
0JH808 Defibrillator Generator
0JH809 Cardiac Resynchronization Defibrillator Pulse Generator
0JH808Z Insertion of Defibrillator Generator into Abdomen Subcutaneous Tissue and Fascia, Open Approach
0JH838Z Insertion of Defibrillator Generator into Abdomen Subcutaneous Tissue and Fascia, Percutaneous Approach
02HN4KZ Insertion of Defibrillator Lead into Pericardium, Percutaneous Endoscopic Approach
02HN0KZ Insertion of Defibrillator Lead into Pericardium, Open Approach
02HK3KZ Insertion of Defibrillator Lead into Right Ventricle, Percutaneous Approach
0JH609Z Insertion of Cardiac Resynchronization Defibrillator Pulse Generator into Chest Subcutaneous Tissue and Fascia, Open Approach
0JH639Z Insertion of Cardiac Resynchronization Defibrillator Pulse Generator into Chest Subcutaneous Tissue and Fascia, Percutaneous Approach
0JH809Z Insertion of Cardiac Resynchronization Defibrillator Pulse Generator into Abdomen Subcutaneous Tissue and Fascia, Open Approach
0JH839Z Insertion of Cardiac Resynchronization Defibrillator Pulse Generator into Abdomen Subcutaneous Tissue and Fascia, Percutaneous Approach
0JH638 Defibrillator Generator
0JH638Z Insertion of Defibrillator Generator into Chest Subcutaneous Tissue and Fascia, Percutaneous Approach
0JH639 Cardiac Resynchronization Defibrillator Pulse Generator
0JH838 Defibrillator Generator
0JH838Z Insertion of Defibrillator Generator into Abdomen Subcutaneous Tissue and Fascia, Percutaneous Approach

© 2021 de Alcantara Lima N et al. *JAMA Network Open.*

Heart Transplant
02YA0Z2 Transplantation of Heart, Zooplastic, Open Approach
02YA0Z1 Transplantation of Heart, Syngeneic, Open Approach
02YA0Z0 Transplantation of Heart, Allogeneic, Open Approac

Circulatory support devices Placement
02RK0JZ Replacement of Right Ventricle with Synthetic Substitute, Open Approach
2RL0JZ Replacement of Left Ventricle with Synthetic Substitute, Open Approach
02WA0JZ Revision of Synthetic Substitute in Heart, Open Approach
02WA0JZ Revision of Synthetic Substitute in Heart, Open Approach
02HA0RS Insertion of Biventricular Short-term External Heart Assist System into Heart, Open Approach
02HA3RS Insertion of Biventricular Short-term External Heart Assist System into Heart, Percutaneous Approach
02HA4RS Insertion of Biventricular Short-term External Heart Assist System into Heart, Percutaneous Endoscopic Approach
5A02116 Assistance with Cardiac Output using Other Pump, Intermittent
5A02216 Assistance with Cardiac Output using Other Pump, Continuous
5A02110 Assistance with Cardiac Output using Balloon Pump, Intermittent
5A02210 Assistance with Cardiac Output using Balloon Pump, Continuous
02HA0RZ Insertion of Short-term External Heart Assist System into Heart, Open Approach
02HA3RZ Insertion of Short-term External Heart Assist System into Heart, Percutaneous Approach
02HA4RZ Insertion of Short-term External Heart Assist System into Heart, Percutaneous Endoscopic Approach
5A02116 Assistance with Cardiac Output using Other Pump, Intermittent
5A02216 Assistance with Cardiac Output using Other Pump, Continuous
02WA0QZ Revision of Implantable Heart Assist System in Heart, Open Approach
02WA0RS Revision of Biventricular Short-term External Heart Assist System in Heart, Open Approach
02WA0RZ Revision of Short-term External Heart Assist System in Heart, Open Approach
02WA3QZ Revision of Implantable Heart Assist System in Heart, Percutaneous Approach
02WA3RS Revision of Biventricular Short-term External Heart Assist System in Heart, Percutaneous Approach
02WA3RZ Revision of Short-term External Heart Assist System in Heart, Percutaneous Approach
02WA4QZ Revision of Implantable Heart Assist System in Heart, Percutaneous Endoscopic Approach
02WA4RS Revision of Biventricular Short-term External Heart Assist System in Heart, Percutaneous Endoscopic Approach
02WA4RZ Revision of Short-term External Heart Assist System in Heart, Percutaneous Endoscopic Approach
02HA0RZ Insertion of Short-term External Heart Assist System into Heart, Open Approach
02HA4RZ Insertion of Short-term External Heart Assist System into Heart, Percutaneous Endoscopic Approach
5A02116 Assistance with Cardiac Output using Other Pump, Intermittent
5A02216 Assistance with Cardiac Output using Other Pump, Continuous
02HA0RZ Insertion of Short-term External Heart Assist System into Heart, Open Approach
02HA4RZ Insertion of Short-term External Heart Assist System into Heart, Percutaneous Endoscopic Approach

© 2021 de Alcantara Lima N et al. *JAMA Network Open.*

5A02116 Assistance with Cardiac Output using Other Pump, Intermittent
5A02216 Assistance with Cardiac Output using Other Pump, Continuous
5A1D90Z Performance of Urinary Filtration, Continuous, Greater than 18 hours Per
5A1D80Z Performance of Urinary Filtration, Prolonged Intermittent, 6-18 hours Per Day
5A1D70Z Performance of Urinary Filtration, Intermittent, Less than 6 Hours Per Day
02HA0RJ Insertion of Short-term External Heart Assist System into Heart, Intraoperative, Open Approach
02HA3RJ Insertion of Short-term External Heart Assist System into Heart, Intraoperative, Percutaneous Approach
02HA4RJ Insertion of Short-term External Heart Assist System into Heart, Intraoperative, Percutaneous Endoscopic Approach
5A02116 Assistance with Cardiac Output using Other Pump, Intermittent
5A0211D Assistance with Cardiac Output using Impeller Pump, Intermittent
A02216 Assistance with Cardiac Output using Other Pump, Continuous
A0221D Assistance with Cardiac Output using Impeller Pump, Continuous

37.66Insertion of implantable heart assist system
37.68Insertion of percutaneous external heart assist device
39.65Extracorporeal membrane oxygenation [ECMO]

© 2021 de Alcantara Lima N et al. *JAMA Network Open.*
